# Design and Characterization of an Equibiaxial Multi-Electrode Dielectric Elastomer Actuator

**DOI:** 10.3390/ma18081693

**Published:** 2025-04-08

**Authors:** Simon Holzer, Bhawnath Tiwari, Stefania Konstantinidi, Yoan Civet, Yves Perriard

**Affiliations:** 1 Integrated Actuators Laboratory (LAI), Ecole Polytechnique Fédérale de Lausanne, Rue de la Maladière 71b, 2000 Neuchâtel, Switzerlandstefania.konstantinidi@epfl.ch (S.K.); yoan.civet@epfl.ch (Y.C.); yves.perriard@epfl.ch (Y.P.); 2 Center for Artificial Muscles (CAM), Ecole Polytechnique Fédérale de Lausanne, Rue de la Maladière 71b, 2000 Neuchâtel, Switzerland

**Keywords:** dielectric elastomer actuator, design of experiments, strain optimization, multi-electrode actuator, soft actuator

## Abstract

With the ongoing journey of automation advancements and a trend towards miniaturization, the choice of actuator plays a crucial role. Over recent years, soft actuators have demonstrated their usefulness in various applications, especially where light weight and high strain are required. Dielectric elastomer actuators (DEAs) are a class of soft actuators that provide high-strain actuation possibilities in applications like biomedicine, logistics, or consumer electronics. A variety of work featuring DEAs for actuation has been carried out in recent years, but a single work detailing the design conception, fabrication, modeling and experimental validation is lacking, especially in the context of achieving high strains with the integration of multiple electrodes and their interaction. This work discusses these issues with an equibiaxial DEA, enabling optimized equibiaxial strain patterns due to full use of the available actuation area. The developed DEA can achieve an equibiaxial strain of 12.75% for actuation at 60 V μm^−1^ over an active area of 7 cm^2^ which is an improvement of 1.3 times compared to traditional dot actuators. These properties position the device as a promising alternative for various applications like cell cultures or microassembly and provide an advantage of optimized use of passive regions within the actuator.

## 1. Introduction

Over recent years, the automation of different industrial tasks has shown advantages, especially in terms of higher throughput combined with improved quality [1]. Actuators with high efficiency, durability and reliability, together with necessary safety standards, are highly demanded. They can be categorized into different groups based on their operating principles, such as electric, pneumatic or hydraulic actuators. The optimal choice of actuator often depends on the properties needed for a certain application. Such properties may be the force outcome [2], actuation speed [3] or simply a miniaturization of the existing versions [4]. Soft actuators provide advantages in applications where flexibility and stretchability are needed compared to traditional actuators. Areas of interest include wearables [5] as well as biomedical applications [6]. A special category of soft actuators is dielectric elastomer actuators (DEAs), consisting of a dielectric material sandwiched between two elastic electrodes [7,8]. Electrostatic actuation of DEAs induces Maxwell stress, which leads to a deformation of the actuator [9]. Compared to other soft actuators, DEAs offer advantageous properties such as higher stretchability [10], fast actuation [11] and low power consumption [12]. In combination with an intelligent design, they can be easily miniaturized and are useful for various applications like in haptic devices to enhance the user experience [13,14], in consumer electronics [15] to enable new applications and in biomedical applications [16] as their energy density is comparable to human muscles. In industrial applications, DEAs bring the advantage of high strain along with their compliant properties (e.g., Dätwyler Holding AG https://datwyler.com/ (accessed on 6 April 2025), mateligent iDEAs GmbH https://ideas.mateligent.de/ (accessed on 6 April 2025)), making them useful in different fields such as the automobile industry. However, to enable these conditions and improve the actuation of DEAs for certain applications, different parameters of the DEA need to be optimized. In the literature, the focus lies normally on optimizing one parameter at a time to improve actuator behavior. Dominant parameters to consider for the optimization include the electrode shape [17,18,19], electrode material [20], dielectric material [21,22] or the applied pre-stretch [23,24,25]. The different studies are compared in the Supplementary File (Table S1). Simultaneous optimization of different parameters and consideration of their interactions are often overlooked. There is a strong need to consider these dominant parameters along with their possible coupling/interaction for the formulation of an optimized actuation system. With a good experimental strategy, such as design of experiments (DOE), this need can be met while reducing the number of experiments and required time [26,27].

In this work, we address these challenges. In Section 2, the design of the DEA and its analysis strategy are presented followed by the introduction of a model describing the strain behavior of the DEA, with the aim to identify the main parameters. Afterwards, the fabrication process is presented, the experimental setup is explained and the statistical design is introduced. Subsequently, the results of the analytical calculation and the experimental validation are presented in Section 3 and analyzed with the statistical designs. In addition, an analysis of the strain behavior of the ring actuators is performed and the electrical interaction between the two electrodes is investigated. Further on, the two devices with the highest strains are characterized by using different input voltages and fatigue testing is performed. The work is summarized at the end in Section 4.

## 2. Material and Methods

### 2.1. Design and Strategy

In this study, an equibiaxial planar DEA (EBDEA) is used. Different design factors are chosen for the analysis of the EBDEA with a design of experiments (DOE) approach. These parameters are the dielectric elastomer thickness *T*, electrode diameter *D* and pre-stretch *P*. In addition, the strain is measured at different electric field strengths *E*. The exact parameter values analyzed during the experiment and the naming of the investigated EBDEAs are summarized in Table 1.

**Figure 1:**
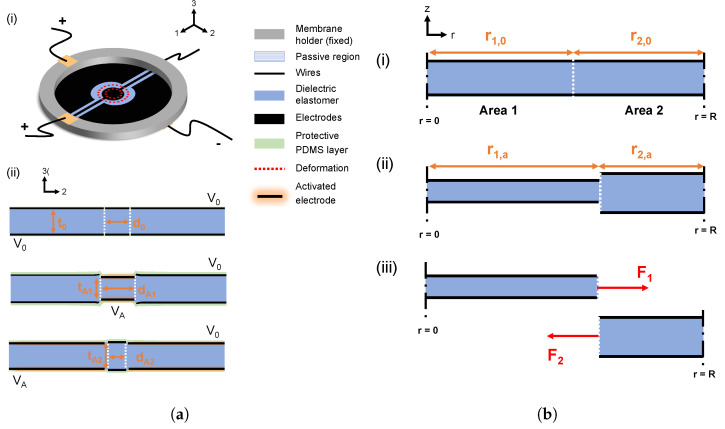
(**a**) (i) Design of the EBDEA including the possible deformation. (ii) Functionality of the EBDEA. By applying a high voltage $V_{A}$ on the disc electrode, tensile stress is induced in the center. By actuating the ring electrode, compressive stress is generated. (**b**) Illustration of the modeling of the EBDEA. (i) The EBDEA before actuation. (ii) The disc electrode is actuated on the EBDEA, leading to a changed ratio between the radii of the two electrodes. (iii) Free-body principle applied on EBDEA.

As presented in Table 1, the EBDEAs are pre-stretched by 1.15 or 1.3 and then clamped between two ring frames with an inner diameter of 30 mm and a outer diameter of 40 mm. The precise values for the pre-stretch are chosen to be in the range used in the literature for other DEA designs. The device design is shown in Figure 1a(i).

The EBDEA consists of two electrodes on each plane: a disc electrode which is surrounded by a ring-shaped electrode to ensure a homogeneous, equibiaxial strain field with maximum strain. The disc electrodes have an unstretched diameter $d_{0}$ of 5 mm or 10 mm. Thus, the diameter of the ring electrode is a sixth and a third of the total dielectric elastomer diameter, respectively. The ring electrodes have an inner diameter of 7 mm or 12 mm so that there is always a distance of around 1 mm between the two electrodes, which is used for insulation to prevent an electrical breakdown between both electrodes. Wires are connected to the electrodes for the necessary power supply. The thickness of the dielectric elastomer is 50 μm or 100 μm. The different thickness are predefined due to the available material thicknesses of the dielectric elastomer. A schematic representation of the behavior of the EBDEA when applying a voltage is presented in Figure 1a(ii). Depending on which electrode the voltage is applied to, a tensile or compressive stress is achieved at the center of the EBDEA.

To determine an ideal design for maximum strain, a full factorial design ($2^{4}$) is applied to the theoretical and measured data of the different EBDEAs. The advantage of the full factorial design is that, besides the influence of the factors on the response, the influence of their interactions can also be analyzed. Thus, factorial designs enable more insight then the traditionally used one-factor-a-time experiments [28]. To analyze the analytical and measured data, a linear model without interaction and with interactions is used similar to [27,29]. The models are defined by the following equations:

Without interactions

(1) $$Y=a_{0}+\sum_{i=1}^{4}a_{i}x_{i},$$

and with interactions

(2) $$Y=a_{0}+\sum_{i=1}^{4}a_{i}x_{i}+\sum_{i<j}^{4}a_{ij}x_{i}x_{j}.$$

$a_{0}$ is the intercept, $a_{i}$ are the half-effect coefficients and $a_{ij}$ are the half-effects of the factor interactions.

### 2.2. Model of Multi-Electrode DEA

In order to determine the behavior of the EBDEA and to compare the different designs with each other, an analytical model is developed. The following assumptions are used for the model:

1. The dielectric material is incompressible: $\lambda_{1}\lambda_{2}\lambda_{3}=1$.
2. The dielectric elastomer actuator is equibiaxially pre-stretched: $\lambda_{1}=\lambda_{2}$.
3. The total radius *R* is constant and is always a sum between the radius of the disc electrode $r_{1}$ and the radial length of the ring electrode $r_{2}$: $r_{1}+r_{2}=R$.

A simplified cross-section along the radial length of the EBDEA is given in Figure 1b. In the following, the radius will be written as $r_{i,j}$, where *i* will stand for the electrode (1 or 2) and *j* will give information on whether the electrode is non-activated (0) or activated (*a*). The strain will be used as $\lambda_{i,j}$, where *i* is used for the electrode (1 or 2) and *j* stands for the direction of the principal strain (1, 2). It is important to mention that these radii are already the pre-stretch corrected values and not the initial fabricated ones. With the help of assumption (3), the following equation is valid:

(3) $$r_{1,0}+r_{2,0}=r_{1,a}+r_{2,a}=R,$$

where $r_{1,0}$ is the radius of the non-actuated disc electrode, $r_{2,0}$ is the radius of the non-actuated ring electrode, $r_{1,a}$ is the radius of the actuated disc electrode and $r_{2,a}$ is the radius of the actuated ring electrode. Therefore, the following two equations can be defined:

(4) $$r_{1,a}=\lambda_{1,1}\;\cdot \;r_{1,0},$$

where $\lambda_{1,1}$ is the strain in area 1 (Figure 1b(i)) and

(5) $$r_{2,a}=\lambda_{2,1}\;\cdot \;r_{2,0},$$

where $\lambda_{2,1}$ is the strain in area 2 (Figure 1b(i)). With the help of these definitions, the following relation between $\lambda_{1,1}$ and $\lambda_{2,1}$ is found:

(6) $$\lambda_{2,1}=1+k\left(1-\lambda_{1,1}\right),$$

where $k=\frac{r_{1,0}}{r_{2,0}}$ is the initial ratio between the radius of the disc electrode and the ring electrode. With the help of this equation, we obtain a connection between the strain of the active and passive parts of the EBDEA. We assume that if the EBDEA is actuated with a constant voltage, a stable equilibrium is established. Thus, we can cut along the thickness of the EBDEA and obtain an equilibrium of force which is shown in Figure 1b(iii). This can be presented by the following equation:

(7) $$F_{1}=F_{2}$$

where $F_{1}$ is the force effects on area 1 and $F_{2}$ is the force effects on area 2. For the simplification, we assume that the thickness of the element is always constant along the whole area *i* (*i* = 1,2). In addition, we simplify the transitional area by having a jump in thickness. Thus, Equation (7) can be rewritten as

(8) $$\sigma_{1}A_{1}=\sigma_{2}A_{2}$$

where $\sigma_{1}$ is the stress in element 1, $A_{1}$ is the cross-section from element 1, $\sigma_{2}$ is the stress in element 2 and $A_{2}$ is the cross-section from element 2.

There are various models for the mechanical description of dielectric elastomers that attempt to describe the non-linear stress–strain behavior. In this study, the Yeoh model approach is used to describe the strain energy density. It is defined as:

(9) $$W_{s}=\sum_{i=1}^{3}C_{i}{\left(I_{1}-3\right)}^{i}$$

where $C_{1}$, $C_{2}$ and $C_{3}$ are the Yeoh coefficients depending on the specific material and $I_{1}$ the first strain invariant of the Cauchy–Green deformation tensor. In this case, the Yeoh parameters for the dielectric material are taken from [30]. They used the same material in the equibiaxial state as in this study and obtained the parameter for Elastosil 2030 from the supplier. A detailed introduction of the model with differentiation of different stress states can be found in Suo [31]. Using this approach, we can write the existing stresses as follows:

(10) $$\begin{array}{cc} \sigma_{1} & =\lambda_{1,1}\frac{\partial (W_{s})}{\partial \lambda_{1,1}}-\varepsilon_{0}\varepsilon_{r}E_{1}+\lambda_{p,1}\frac{\partial (W_{s,p})}{\partial \lambda_{p,1}}\\ \; & =2\left(\lambda_{1,1}^{2}-\lambda_{1,1}^{-4}\right)(C_{1}+2C_{2}\left(2\lambda_{1,1}^{2}+\lambda_{1,1}^{-4}-3\right)\\ \; & +3C_{3}{\left(2\lambda_{1,1}^{2}+\lambda_{1,1}^{-4}-3\right)}^{2})-\varepsilon_{0}\varepsilon_{r}\lambda_{1,1}^{4}\frac{V_{d}^{2}}{t_{0}^{2}}\\ \; & +2\left(\lambda_{p,1}^{2}-\lambda_{p,1}^{-4}\right)(C_{1}+2C_{2}\left(2\lambda_{p,1}^{2}+\lambda_{p,1}^{-4}-3\right)\\ \; & +3C_{3}{\left(2\lambda_{p,1}^{2}+\lambda_{p,1}^{-4}-3\right)}^{2}), \end{array}$$

where $\lambda_{1,1}$ is the principal strain in area 1, $W_{s}$ is the strain energy density, $\varepsilon_{0}$ is the vacuum permittivity, $\varepsilon_{r}$ is the relative permittivity, *E* is the electric field which is defined by the applied voltage on the disc electrode $V_{d}$ and the thickness is $t_{a}=\lambda_{1,1}t_{0}$; $t_{0}$ is thereby the initial thickness of the EBDEA. The stress $\sigma_{2}$ is defined in a similar way, except that the stretch is adapted and the actuation voltage of the ring electrode is used:

(11) $$\begin{array}{cc} \sigma_{2} & =\lambda_{2,1}\frac{\partial (W_{s})}{\partial \lambda_{2,1}}-\varepsilon_{0}\varepsilon_{r}E_{2}+\lambda_{p,1}\frac{\partial (W_{s,p})}{\partial \lambda_{p,1}}\\ \; & =2\left(\lambda_{2,1}^{2}-\lambda_{2,1}^{-4}\right)(C_{1}+2C_{2}\left(2\lambda_{2,1}^{2}+\lambda_{2,1}^{-4}-3\right)\\ & +3C_{3}{\left(2\lambda_{2,1}^{2}+\lambda_{2,1}^{-4}-3\right)}^{2})-\varepsilon_{0}\varepsilon_{r}\lambda_{2,1}^{4}\frac{V_{r}^{2}}{t_{0}^{2}}\\ \; & +2\left(\lambda_{p,1}^{2}-\lambda_{p,1}^{-4}\right)(C_{1}+2C_{2}\left(2\lambda_{p,1}^{2}+\lambda_{p,1}^{-4}-3\right)\\ \; & +3C_{3}{\left(2\lambda_{p,1}^{2}+\lambda_{p,1}^{-4}-3\right)}^{2}), \end{array}$$

where $\lambda_{2,1}$, the first principal strain in area 2, is defined by Equation (6). The cross-sections for the model are given by

(12) $$\begin{array}{cc} A_{1} & =2\pi r_{1,a}t_{a,1}=2\pi r_{1,0}\lambda_{1,1}\frac{t_{0}+2t_{e}}{{\left(\lambda_{1,1}\right)}^{2}}\\ \; & =\frac{2\pi r_{1,0}\left(t_{0}+2t_{e}\right)}{\lambda_{1,1}}, \end{array}$$

where $t_{a,1}$ is the actuated thickness of the disc electrode, which includes the original dielectric elastomer thickness $t_{0}$ and two times the electrode thickness $t_{e}$ on which the stretch is applied:

(13) $$t_{a_{1}}=\frac{t_{0}+2t_{e}}{\lambda_{1,1}^{2}}.$$

The same is valid for cross-section $A_{2}$:

(14) $$\begin{array}{cc} A_{2} & =2\pi r_{1,a}t_{a,2}=2\pi r_{1,0}\lambda_{1,1}\frac{t_{0}+2t_{e}}{{\left(\lambda_{2,1}\right)}^{2}}\\ \; & =2\pi r_{1,0}\left(t_{0}+2t_{e}\right)\frac{\lambda_{1,1}}{1+k\left(1-\lambda_{1,1}\right){)}^{2}}, \end{array}$$

where $t_{a,2}$ is the actuated thickness of the ring electrode defined in the same way as $t_{a_{1}}$. The cross-section $A_{2}$ is directly given by the $\lambda_{1,1}$. By putting all these terms together, a relation between the strain and the applied voltage is realized. These relations are valid for the actuation of the disc as well as the ring electrode, depending on the chosen actuation voltage $V_{i}$ (i = d,c).

### 2.3. Fabrication

**Figure 2:**
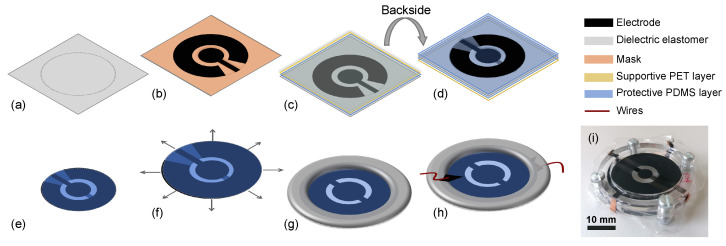
Fabrication of EBDEA: (**a**) Preparation of dielectric elastomer sheet with chosen thickness. (**b**) Application of the first electrode on the surface of the DE. (**c**) Application of a very thin layer of PDMS (Sylgard 186, Sigma-Aldrich, St. Louis, MO, USA) on the surface to glue a protective PET sheet onto the dielectric elastomer surface. (**d**) Flipping the EBDEA from previous stage to the other side and removing the protective PET layer from the second side. Subsequently, the second electrode is printed on the DE. (**e**) Cutting of the EBDEA using a laser cutter. (**f**) Application of pre-stretch. (**g**) Gluing of an acrylic glass frame to hold the material in its stretched position. (**h**) Wire bonding of the EBDEA. (**i**) Fabricated EBDEA.

The objective of the fabrication is to have a two-layer DEA with a disc- and ring-shaped electrode. For the fabrication, an elastomer film (Elastosil 2030, Wacker Chemie AG, Stuttgart, Germany) with a thickness of 50 μm and 100 μm is used (Figure 2a). The maximum thickness variation across the total width is less than ±5%. The relative permittivity for the elastomer film is 2.8 and the electrical breakdown field varies between 80 V μm^−1^ to 100 V μm^−1^. The carbon composite electrodes are applied onto the dielectric elastomer (Figure 2b) using a film applicator (ZAA2300, Zehntner GmbH Testing Instruments, Sissach, Switzerland). A detailed description and characterization of the used electrodes are presented in [32]. In the next step, a thin layer of polydimethylsiloxane (PDMS) is applied to the whole dielectric elastomer surface and a polyethylene terephthalate (PET) sheet is placed on the thin PDMS film (Figure 2c). The thin layer of PDMS provides necessary insulation of the electrodes from the environment. After repeating the curing in an oven with the same parameters, the original PET protective layer is removed and the process is repeated for the other side of the dielectric elastomer (Figure 2d). Upon electrode fabrication, the thickness of the electrode is measured using a laser confocal microscope (Keyence VK X-1000, Keyence International NV/SA, Mechelen, Belgium) and is found to be around 20μm. In the next step, the fabricated structure is patterned in a round shape using a laser cutter (Figure 2e). Then, the whole elastomer is equibiaxially pre-stretched (Figure 2f) using a custom-made setup (Section 2.4). With the help of the setup, the uniformity of the pre-stretch is ensured and devices without uniform pre-stretch are sorted out (i.e., due to release of membrane). Afterwards, an acrylic frame with an inner diameter of 30 mm is glued onto the stretched membrane to maintain the pre-stretch post-fabrication (Figure 2g). Using conductive silicone (Elastosil LR 3162, Wacker Chemie AG, Stuttgart, Germany) and copper tape, the wires are bonded to the electrodes (Figure 2h).

### 2.4. Setups

**Figure 3:**
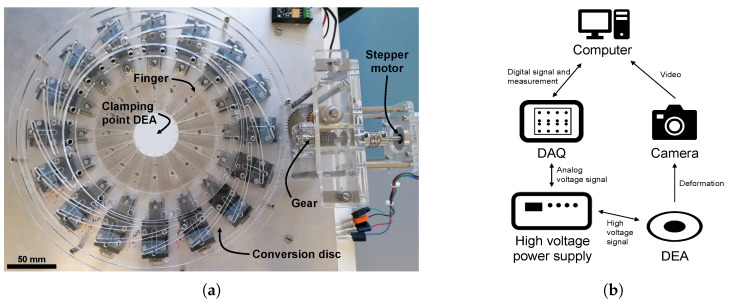
(**a**) Pre-stretch setup: EBDEA is clamped on the center of the sixteen fingers. By actuating the stepper motor, a movement is transferred into radial movement of the fingers by the gear and the conversion disc. (**b**) Working schematics of the experimental setup.

For the application of equibiaxial pre-stretch, a custom-made setup is employed, featuring sixteen fingers moving in the radial direction (Figure 3a). This arrangement ensures a quasi-homogeneous pre-stretching of the dielectric elastomer in the radial direction. The pre-stretch setup is equipped with a camera (Canon EOS D60, Canon Inc., Tokyo, Japan) capturing images, facilitating precise determination of the pre-stretch and its uniformity for each EBDEA. The precise pre-stretch of the EBDEA is determined with image processing.

For the actuation of the EBDEA, a setup including a DAQ (USB X Series Multifunction DAQ, National Instruments, Austin, TX, USA) and a high power supply (Trek 10/40A-HS, Advanced Energy, Denver, CO, USA) is used. During the actuation, the frames constituting deformation are acquired using a camera (Canon EOS 650D, Canon Inc., Tokyo, Japan) and analyzed with MATLAB R2022b (The MathWorks Inc., Natick, MA, USA). Figure 3b shows a schematic illustration of the described experimental setup.

## 3. Results and Discussion

In the first part, the results of the optimization of the different design factors for the EBDEA with multiple electrodes are discussed. This includes a comparison between the model and experimental data and a discussion about the models determined using the analysis with the full factorial design. In a further step, the strain field generated by the actuator is discussed in more detail. The focus is placed on the inhomogeneous behavior of the ring electrodes, which is caused by the connections of the disc electrodes. Furthermore, the two best performing designs in terms of the maximum strain are compared and characterized. A step signal and triangular actuation signal are used to gain more insight into the actuator behavior. In addition, multiple cycles (200 step signals) are presented to show the evolution of the deformation for constant step input voltages.

### 3.1. Strain Optimization with Full Factorial Design

**Figure 4:**
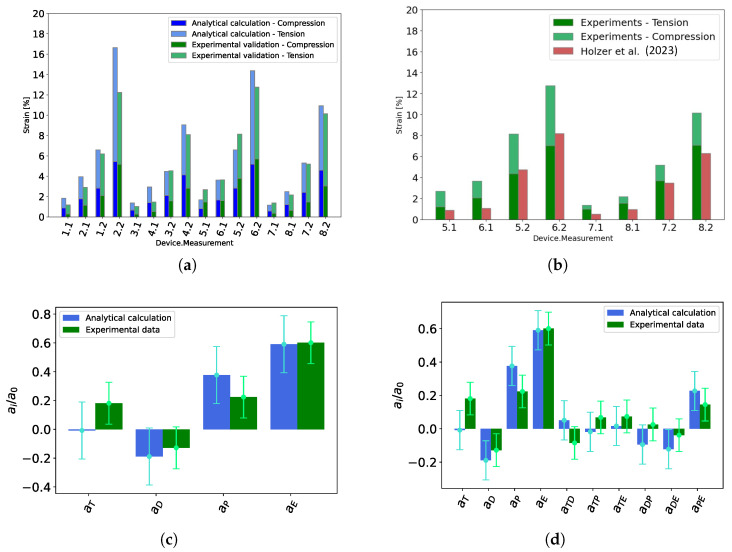
(**a**) Total strain of analytical calculation and experimental validation divided into compressive and tensile strain. (**b**) Comparison of achievable strain by using just a disc electrode or combined disc–ring electrodes [29]. (**c**) Relative half-effects for a linear model withoutinteractions given for the data determined with analytical calculation and experimental validation. (**d**) Relative half-effects for a linear model with interactions given for the data determined with analytical calculation and experimental validation.

The strain values achieved by the analytical model and the experiments are given in Table 2 and in Figure 4a. The EBDEAs distinguish themselves by the design (Section 2.1) and they are actuated at 30 V μm^−1^ and 60 V μm^−1^. Thus, eight EBDEAs with different designs are used for the data (see Table 1).

The calculated values (Table 2) predict higher strains than the experimental data. To simplify the calculations, the presented study considered negligible influence of the transition areas of the electrodes, the connecting area (for disc electrodes) and the dependent areas around the outer electrodes’ periphery near to the clamping frame, leading to this overestimation. The effect of the inhomogeneity due to the electrodes’ connections to the circular electrodes is also not included in the model and leads to further overestimation. The inhomogeneity is further analyzed in Section 3.3. In a next step, the generated data is analyzed using the full factorial model ($2^{4}$). The analysis is carried out for both the theoretical and the measured data. The result for the model without interactions is shown in Figure 4c. The pre-stretch and the actuation with the electric field are statistically relevant, which means that an increase in these values also leads to an increase in deformation. For the thickness of the dielectric elastomer, the model without interactions shows that the data obtained with the analytical model do not lead to an improvement. In contrast, only an increase in thickness increases the deformation as well. This difference is assumed to occur due to the assumptions which are used for the analytical model. In the case of deformation, the smaller the diameter of the disc electrode, the greater the possible deformation. However, in both cases the confidence interval is so large that this is not statistically relevant. In a second step, the same data for the analytical model and the experiment is also analyzed using a model with interaction (Figure 4d). The information obtained from the model with interactions is similar for the factors *T*, *P* and *E* as for the model without interactions. For factor D, the diameter of the electrodes, the confidence interval is not crossing zero, leading to a clear statistical prediction of the behavior by changing the diameter. In general, it is important to note that the diameter of the electrode is certainly limited to a certain minimum size, which will be defined by the application and the required actuation area. As far as interactions are concerned, none are really of great importance, except the interaction DE in the case of analytical data and the interaction PE in the case of both data. This means, by changing one of these values, the interaction term can enhance or reduce the wanted effect. The analyzed models can be written as formulas. This results in the following formulas:

Analytical data and model without interactions:

(15) $$\begin{array}{cc} y= & a_{0}+a_{P}x_{P}+a_{E}x_{E}\\ = & 5.81+2.19x_{P}+3.43x_{E}. \end{array}$$

Experimental data and model without interactions:

(16) $$\begin{array}{cc} y= & a_{0}+a_{T}x_{T}+a_{P}x_{P}+a_{E}x_{E}\\ = & 4.87+0.88x_{T}+1.09x_{P}+2.93x_{E}. \end{array}$$

Analytical data and model with interactions:

(17) $$\begin{array}{cc} y= & a_{0}+a_{D}x_{D}+a_{P}x_{P}+a_{E}x_{E}\\ & +a_{D}Ex_{D}E+a_{P}Ex_{P}E\\ = & 5.81-1.10x_{D}+2.19x_{P}+3.43x_{E}\\ & -0.71x_{D}E+1.32x_{P}E. \end{array}$$

Experimental data and model with interactions:

(18) $$\begin{array}{cc} y= & a_{0}+a_{T}x_{T}+a_{D}x_{D}+a_{P}x_{P}\\ & +a_{E}x_{E}+a_{P}Ex_{P}E\\ = & 4.87+0.88x_{T}-0.63x_{D}+1.09x_{P}\\ & +2.93x_{E}+0.71x_{PE}. \end{array}$$

### 3.2. Comparison of Actual Design with Design Excluding Ring Electrode

The new design, with a disc and ring electrode combined, is intended to provide an improvement in terms of maximum deformation compared to the standard dot actuator, for example, as discussed in [29,30]. To compare the difference between the two designs, the data measured for a thickness of 100 μm is compared with the data from [29]. The devices in the previous study are fabricated in the same way and differ only in the additional ring electrodes and a small mismatch in the pre-stretch, 12.5 vs. 15% and 25 vs. 30%. In Figure 4b, a comparison between the data is provided. The maximum strain can be improved by adding the second electrode. This is in line with expectations, as the active area has increased significantly in the new design. The values of the disc electrodes are relatively similar for the old and the new design and no clear tendency is visible. This is consistent with the expectations, as only the addition of the ring electrodes is influencing the behavior by slightly stiffening the area around the disc electrodes. We assume that this stiffening effect is balanced by the slightly high pre-stretch applied to the new design. All in all, the new design enables a higher maximum strain compared to the old design. This can be attributed to the larger surface area that is now available for deformation compared to the dot actuator. In addition to the advantage of the higher strain, however, the EBDEAs is more complex due to the extra electrode. An additional actuation signal needs to be generated, which will control the second electrode. This actuation signal must be independent of the actuation signal of the other electrode. In addition, the electrodes tend to interact with each other and this can lead to an electrical breakdown between them, which may destroy the EBDEA.

### 3.3. Analysis of Inhomogeneous Compressive Strain

**Figure 5:**
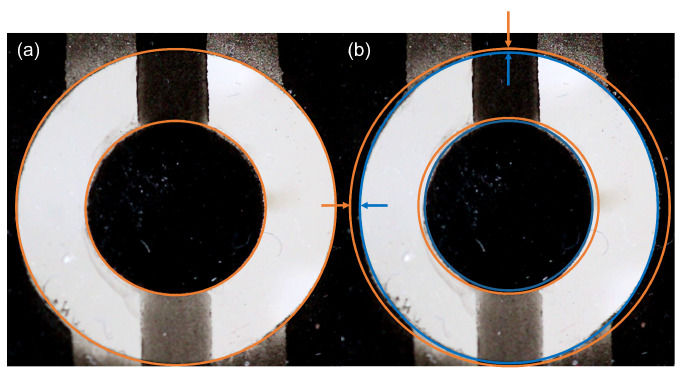
Inhomogeneous actuation of the ring electrode. (**a**) Electrodes before actuation. (**b**) Electrodes after actuation of the ring electrode (blue) are deformed inhomogeneously compared to electrodes without actuation (orange).

Due to the connectors of the disc electrodes, the ring electrodes cannot be completely closed. As a result, the outer electrode corresponds to two unclosed cs rather than a whole ring (Figure 1a). As a result, the actuation of the ring electrode leads to an inhomogeneous strain. This inhomogeneity is shown in Figure 5. A typical actuation with 60 V μm^−1^ leads to about 6.7% and 3.5% deviation in strain, respectively, along the *x* and *y* axes because of the mentioned presence of inhomogeneity. This may be minimized by improving the fabrication and connector patterning.

### 3.4. Characterization of EBDEAs

**Figure 6:**
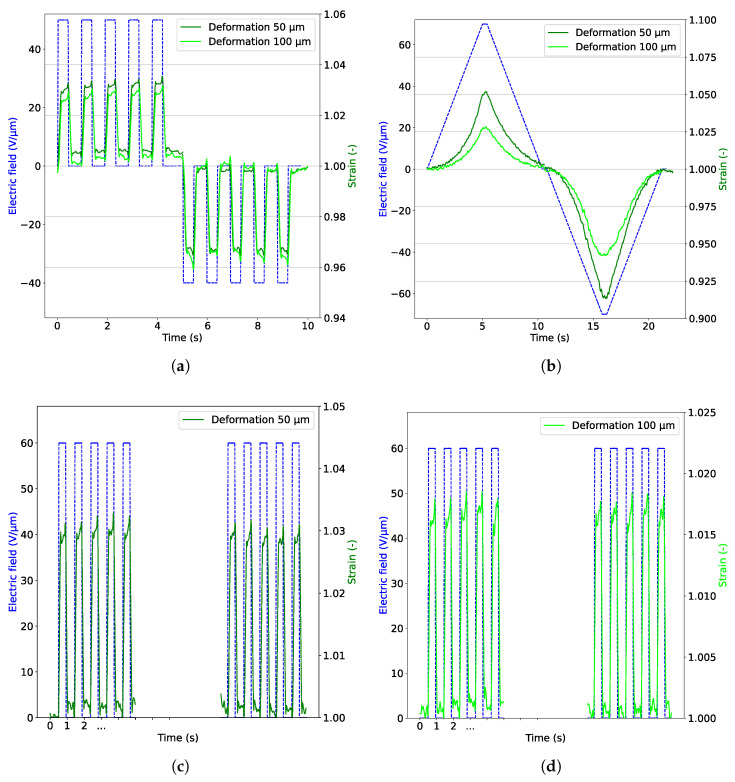
(**a**) Step actuation of the two EBDEAs shows differences in their dielectric thickness (50/100 μm) for an electric field of 25 V μm^−1^ for the disc electrodes and 20 V μm^−1^ for the ring electrodes. (**b**) Triangular actuation of the two EBDEAs with respective dielectric thickness of 50 and 100 μm. (**c**) Repeatability test over 200 actuation cycles of EBDEA 2. Cycles 1–5 and 196–200 are shown in the figure. (**d**) Repeatability test over 200 actuation cycles of EBDEA 6. Cycles 1–5 and 196–200 are shown in the figure.

By analyzing the experimental data, EBDEAs 2 and 6 are the most promising ones to achieve maximum strain. Therefore, characterization of the two EBDEAs is presented in this section. First, the EBDEAs are actuated with a step voltage to show their dynamic behavior. In Figure 6a, their actuation with a step input is plotted. The disc electrodes of the two EBDEAs are actuated with 50 V μm^−1^ and the ring electrodes with 40 V μm^−1^.

The tensile deformation is slightly increased in the 50 μm thick material, whereas the compression is slightly better in the 100 μm thick material. The strain needs a moment to decrease again when a tensile strain is activated. This is due to the viscoelastic material behavior of Elastosil 2030. Interestingly, however, the effect is greater in the tensile range than in the compressive one. What is also apparent is that the compression causes the viscoelastic effect to practically disappear after the material has been tensile-stretched. This means that the design of the electrodes has created an actuator with which the viscoelastic material behavior can be better controlled. With a suitable control algorithm, it can even be assumed that the viscoelastic behavior can be completely suppressed by a suitable similar actuation of both electrodes. Actuation with a triangular signal gives more information about the static behavior of the EBDEA (Figure 6b). The typically quadratic relationship between the actuation stress and the deformation is visible in the plot. Compared to the measured values at 30 V μm^−1^ and 60 V μm^−1^ where the deformation is quite similar for device 2 and 6, the absolute difference becomes larger due to this quadratic dependence for values higher than 60 V μm^−1^. It means that device 2 generates more strain for high electric fields than device 6. In addition, the charging and discharging behaviors are not completely symmetrical. This is the hysteresis that occurs due to the viscoelastic material behavior of the dielectric elastomer. The dielectric elastomer usually needs a certain amount of time to return to its original position. However, as mentioned before, this phenomenon can be counteracted with the chosen design including disc and ring electrodes. With the help of suitable control and actuation of the two electrodes, the effect can be eliminated. In addition to determining the static and dynamic behavior, it is also important to observe the behavior over several actuation cycles. Therefore, multiple cycles (200 times) are performed. The actuation field is chosen at 600 V μm^−1^, as this value can correspond to a realistic application in which the deformation is optimized on the one hand, but on the other hand a sufficiently large safety margin is taken into account before the electrical breakdown occurs. This number of cycles is chosen because in many applications of DEAs this number is sufficient to cover the entire service life. The outcome of the fatigue testing is shown in Figure 6c,d. For both actuators, there is no difference in the behavior of the actuator after 200 actuation cycles.

## 4. Conclusions

This work presents a novel equibiaxial DEA-based design. In this study, an analytical model of the EBDEA is presented and discussed. In addition, the fabrication and the experimental setup are introduced. Then, several variants of the design are compared with each other using a full factorial model and optimized for maximum strain. Strains of up to and over 12% are shown for an actuation field of 60 V μm^−1^. The obtained strain is 30 percent higher compared to current dot actuators, which usually only achieve actuation of up to 9% for the same dielectric material, its thickness and its actuation field [29]. The two most promising variants are selected and further compared with each other. It is shown that the EBDEA with the thinner dielectric elastomer allows a higher strain, especially at high actuation fields. It is also shown that the EBDEA can withstand a lifetime of 200 cycles without any change in strain. This lifetime is sufficient for many applications, for example, the use of an EBDEA as a cell stretcher [33,34]. The chosen design and a suitable control should prevent hysteresis in the future, which is unique to this design compared to others.

Topology optimization or machine learning-based approaches could be of great interest to decrease inhomogeneity in future work, allowing for a more dedicated design and fabrication strategy.

## Figures and Tables

**Table 1:** Factors, their range of values and nomenclature.

DE Thickness	Electrode Ø	Pre-Stretch	E-Field	Device Measurement
$x_{1}$ [μm]	$x_{2}$ [mm]	$x_{3}$ [-]	$x_{4}$ [V μm^−1^]	(D.M)
50	5/7	1.15	30	1.1
50	5/7	1.30	30	2.1
50	5/7	1.15	60	1.2
50	5/7	1.30	60	2.2
50	10/12	1.15	30	3.1
50	10/12	1.30	30	4.1
50	10/12	1.15	60	3.2
50	10/12	1.30	60	4.2
100	5/7	1.15	30	5.1
100	5/7	1.30	30	6.1
100	5/7	1.15	60	5.2
100	5/7	1.30	60	6.2
100	10/12	1.15	30	7.1
100	10/12	1.30	30	8.1
100	10/12	1.15	60	7.2
100	10/12	1.30	60	8.2

**Table 2:** Compressive, tensile and total strain for analytical calculation and measurements for device (D) and measurement (M): CCS (calculated compressive strain), CTS (calculated tensile strain), TSC (total strain calculated), MCS (measured compressive strain), MTS (measured tensile strain) and TSM (total strain measured).

D.M	CCS	CTS	TCS	MCS	MTS	TMS
1.1	0.87	0.96	1.83	0.30	0.87	1.17
2.1	1.78	2.17	3.95	1.17	1.76	2.04
1.2	2.83	3.76	6.59	2.10	4.10	6.2
2.2	5.45	11.19	16.64	5.17	7.06	7.41
3.1	0.67	0.70	1.37	0.28	0.75	1.03
4.1	1.40	1.54	2.94	0.53	0.93	1.46
3.2	2.11	2.36	4.47	1.57	2.97	4.54
4.2	4.13	4.93	9.06	2.83	5.26	8.09
5.1	0.81	0.87	1.68	1.47	1.21	2.68
6.1	1.66	1.96	3.61	1.59	2.06	3.65
5.2	2.83	3.76	6.59	3.79	4.35	8.09
6.2	5.18	9.19	14.38	5.70	7.05	12.75
7.1	0.57	0.59	1.16	0.36	1.01	1.37
8.1	1.21	1.28	2.49	0.64	1.53	2.17
7.2	2.41	2.88	5.29	1.48	3.71	5.19
8.2	4.58	6.34	10.92	3.05	7.09	10.14

## Data Availability

The raw data supporting the conclusions of this article will be made available by the authors on request.

## Supplementary Materials

The following supporting information can be downloaded at https://www.mdpi.com/article/10.3390/ma18081693/s1: Table S1: Comparison of different approaches to optimize dielectric elastomer actuators (DEAs).

## Abbreviations

The following abbreviations are used in this manuscript:

DEA	Dielectric elastomer actuator
DOE	Design of experiments
EBDEA	Equibiaxial planar dielectric elastomer actuator
PDMS	Polydimethylsiloxane
PET	Polyethylene terephthalate
