# Supplementary material for: Design and Characterization of an Equibiaxial Multi-Electrode Dielectric Elastomer Actuator
Source: Materials (Basel). 2025 Apr 8;18(8):1693. doi: 10.3390/ma18081693 (PMC12028905; doi:10.3390/ma18081693)
Supplement: Supplementary file 1 [file materials-18-01693-s001.zip › materials-3531497-supplementary.pdf]

materials

MDPI

Supplementary Materials

Design and Characterization of an Equibiaxial Multi-Electrode Dielectric Elastomer Actuator

Simon Holzer [1,2*], Bhawnath Tiwari [1,2], Stefania Konstantinidi [1,2], Yoan Civet [1,2] and Yves Perriard [1,2]

[1] Integrated Actuators Laboratory (LAI), Ecole Polytechnique Fédérale de Lausanne, Rue de la Maladière 71b, 2000 Neuchâtel, Switzerland
[2] Center for Artificial Muscles (CAM), Ecole Polytechnique Fédérale de Lausanne, Rue de la Maladière 71b, 2000 Neuchâtel, Switzerland
* Correspondence: simon.holzer@epfl.ch

Table S1. Comparison of different approaches to optimize dielectric elastomer actuators (DEAs).

Title	Year	Author	Optimized Parameter	Notes	Citation
Design of dielectric elastomer actuators using topology optimization on electrodes	2020	Bicheng Chen, Nianfeng Wang, Xianmin Zhang and Wei Chen	Electrode shape	Two actuators are designed and tested for verifications of maximum displacement.	[17]
Geometric optimization of dielectric elastomer electrodes for dynamic applications	2021	Emil Garnell, Bekir Aksoy, Corinne Rouby, Herbert Shea, Olivier Doaré	Electrode shape	FEM to compute optimal design. Optimal design tested afterwards	[18]
Optimal Design of Electrode Topology of Dielectric Elastomer Actuators Based on the Parameterized Level Set Method	2023	Peng Zhang, Ziwen Yan, Kai Luo and Qiang Tian	Electrode Shape	Presentation of two case studies to validate proposed approach. Comparison of numerical results with experimental results.	[19]
Flexible and Stretchable Electrodes for Dielectric Elastomer Actuators	2012	Samuel Rosset and Herbert Shea	Electrode materials	Review on different electrodes used for DEAs. Advantages of different electrode materials are analyzed.	[20]
Evaluation of dielectric elastomers to develop materials suitable for actuation	2021	Philippe Banet, Nouh Zeggai, Jonathan Chavanne, Giao T. M. Nguyen, Linda Chikh, Cédric Plesse, Morgan Almanza, Thomas Martinez, Yoan Civet, Yves Perriard and Odile Fichet	Dielectric material	Analysis of different available dielectric elastomer. Different polymer modification and its influence are screened.	[21]

In silico optimization of actuation performance in dielectric elastomer composites via integrated finite element modeling and deep learning	2024	Jiaxuan Ma and Sheng Sun	Dielectric material (filler content, aspect ratio orientation and position of composites)	Investigation of the impact of doped particles on actuation performance of DE composites. A machine learning framework is used for further advancing DE composite designs.	[22]
Power optimization of a conical dielectric elastomer actuator for resonant robotic systems.	2020	Chongjing Cao, Xing Gao, Stuart Burgess and Andrew Conn	Power	Characterization of the dynamic response with a numerical model. Model used for optimization of power against pre-stretch and spacer length.	[23]
Influence of Axial Pre-stretch on Tubular Dielectric Elastomer Actuators	2022	Amine Benouhiba, Armando Walter, Thomas Martinez, Yoan Civet and Yves Perriard	Pre-stretch	Analysis of the coupling of the internal pressure with pre-stretch to show functioning and stability of DEA based cardiac assist device.	[24]
Performance-Optimized Dielectric Elastomer Actuator System with Scalable Scissor Linkage Transmission	2022	Daniel Bruch, Tobias Pascal Willian, Hendrik Cornelius Schäfer and Paul Motzki	Force and displacement	Combination of buckled beam and DEA by a scissor linkage mechanism. Different designs of the mechanism are validated through experiments.	[25]
Towards Optimal Design of Dielectric Elastomer Actuators Using a Graph Neural Network Encoder	2023	Yangfan Li, Jun Liu, Wenyu Liang and Zhuangjian Liu	Electrode shape	A graph neural network is used to determine target response. Three optimized designs are then fabricated and tested.	[26]
An Experimental Approach for the Design of Uni-axial Fiber Reinforced Dielectric Elastomer Actuators	2023	Stefania Konstantinidi, Julian Asboth, Armando Walter, Simon Holzer, Thomas Martinez, Yoan Civet and Yves Perriard	Fiber width, Fiber thickness and distance between fibers	Fibers are integrated to DEAs to enhance performance. Thereby, the fiber design is optimized.	[27]
Influence of Active-to-Passive Ratio on the Deformation in Circular Dielectric Elastomer Actuators	2024	Markus Koenigsdorff, Hans Liebscher, Petr Osipov, Johannes Mersch	Electrode shape	Introduction of a novel model describing the behavior of a dot DEA. Analysis of the electrode shape	[31]

		and Gerald Gerlach		with the model and experimental results.	
This study	2025	Simon Holzer, Bhawnath Tiwari, Stefania Konstantinidi, Yoan Civet and Yves Perriard	Electrode shape, dielectric elastomer thickness, pre-stretch, actuation voltage	A model is introduced to describe the actuator design. It is afterwards compared with eight actuators to improve the maximum strain.	-
